# Enhancing the Breast Histopathology Image Analysis for Cancer Detection Using Variational Autoencoder

**DOI:** 10.3390/ijerph20054244

**Published:** 2023-02-27

**Authors:** Harsh Vardhan Guleria, Ali Mazhar Luqmani, Harsh Devendra Kothari, Priyanshu Phukan, Shruti Patil, Preksha Pareek, Ketan Kotecha, Ajith Abraham, Lubna Abdelkareim Gabralla

**Affiliations:** 1 Symbiosis Institute of Technology, Symbiosis International University, Pune 412115, India; 2 Faculty of Computing and Data Sciences, FLAME University, Lavale, Pune 412115, India; 3 Department of Computer Science and Information Technology, College of Applied, Nourah Bint Abdulrahman University, Riyadh 11671, Saudi Arabia

**Keywords:** variational autoencoder, histopathology image, deep learning

## Abstract

A breast tissue biopsy is performed to identify the nature of a tumour, as it can be either cancerous or benign. The first implementations involved the use of machine learning algorithms. Random Forest and Support Vector Machine (SVM) were used to classify the input histopathological images into whether they were cancerous or non-cancerous. The implementations continued to provide promising results, and then Artificial Neural Networks (ANNs) were applied for this purpose. We propose an approach for reconstructing the images using a Variational Autoencoder (VAE) and the Denoising Variational Autoencoder (DVAE) and then use a Convolutional Neural Network (CNN) model. Afterwards, we predicted whether the input image was cancerous or non-cancerous. Our implementation provides predictions with 73% accuracy, which is greater than the results produced by our custom-built CNN on our dataset. The proposed architecture will prove to be a new field of research and a new area to be explored in the field of computer vision using CNN and Generative Modelling since it incorporates reconstructions of the original input images and provides predictions on them thereafter.

## 1. Introduction

Breast cancer is indeed a type of cancer that occurs in the cell lines of a woman’s breast. Breast cancer can be classified based on the visual representation of the cells. Ductal Carcinoma in Situ (DCIS) and Invasive Ductal Carcinoma (IDC) are two of the most frequently reported types. DCIS does not affect patients’ everyday lives and accounts for a small fraction of occurrences. IDC is more dangerous and includes the majority of patients [1].

If breast cancer may be found in its earliest stages, then there are many ways to perform a good treatment. As a result, having access to the appropriate screening technologies is crucial for identifying the early signs of breast cancer [2]. Three frequent imaging modalities used to test for this illness include mammography, ultrasonography, and thermography. Mammography [3] is among the most important tools for detecting breast cancer early. Ultrasound or diagnostic sonography treatments are often employed since mammography is ineffective for breasts. Tiny masses can be avoided using radiography, and thermography will be far more efficient than ultrasound in identifying tiny malignant masses [1]. 

Synthetic systems [4], which are neurally utilised in Deep Learning (DL), are an element of the device learning family. Computer eyesight, sound recognition, message recognition, social networking filtering, normal language processing, device interpretation, medication design, materials scrutiny, bioinformatics, and histopathological diagnosis are typical applications of deep learning architectures [5,6]. These brand-new technologies, especially DL algorithms [7], could be used to increase cancer tumour detection precision and effectiveness [8].

The digital pathology (DP) method digitises histology slides in order to generate high-resolution photographs. When using the analysis techniques of images, these digital pictures are employed for detection, segmentation, and classification. To grasp the patterns needed for picture classification, DL with Convolutional Neural Networks (CNNs) requires further stages, such as digital staining. CNNs are a neural network subclass proficient at input processing, for example, using images. Digital pictures are binary representations of visual data. They are made up of a grid-like arrangement of pixels, each of which has a pixel value to indicate how bright and what colour it should be [4].

We must make sure that the latent space is sufficiently regular to use the decoder of our autoencoder for generative purposes. Implementing explicit regularisation throughout the training phase is one way to achieve this regularity. Therefore, training an autoencoder is regularised for overfitting prevention and to ensure that the latent space has favourable features so that the generative processes can be described as a variational autoencoder (VAE).

The VAE architecture combines an encoder–decoder and shrinks the errors produced during reconstruction by using data that are reconstructed and encoded–decoded and the input data [9,10]. The encoding–decoding procedure is somewhat altered as a result. The model is then trained in the following manner. Firstly, over the latent space, the input is encoded. From this space, a point is chosen. The reconstruction error is computed from the decoded sample point. The network undergoes a backpropagation of the reconstruction error.

The issues with the VAE network are related to the learning “identity function” or “null function”. Problems may occur when the output may contain similar values as the input, which tends to affect the autoencoder when the nodes in the hidden layers are greater than the nodes in the input layer. This problem can be solved using Denoising Variational Autoencoder (DVAE) by making false inputs and setting the input to zero. This portion is approximately 30% to 50%, which is further dependent on the number of nodes in the input or data availability [11].

Comparing the output values with the original input rather than the corrupted input when computing the loss function is crucial. By doing so, the chances of extracting features instead of learning the null function are avoided.

This work can be used extensively in situations that deal with healthcare and educational areas. The model’s predictions can be used to obtain important knowledge related to the histopathological classification of breast cancer. In the insurance sector, it can be used to realise the client’s medical situation so that specialists can offer insurance that is specifically tailored to the needs of the customer. In the medical sector, it can be used to predict cancer accurately and well in advance, saving the patient from a potentially fatal condition. Additionally, it reduces the time that pathologists would have to spend manually analysing the report.

### Major Contributions

This research aims to improve the image quality by regenerating histopathological images;

- In this paper, we provide a background study and a new approach to breast cancer detection;
- In the proposed approach, VAE is used to reconstruct images by using CNNs to improve breast cancer detection;
- Histopathological images are processed and presented;
- The prediction results of the proposed approach are provided using different configurations of CNNs with autoencoder variants.

The remaining article is organised as follows: related work and research gaps are present in Section 2. A proposed approach for the implementation is presented in Section 3. The experimental setting is shown in Section 4. Conclusions are presented in Section 5.

## 2. Related Work

According to the literature, reviews of histopathological image analysis, stain normalisation, segmentation, and classification have been conducted by a small number of researchers. In this section, the specifics relating to the overview of the analysis of histopathological images are covered and summarised. The most up-to-date, cutting-edge Computer-aided Design (CAD) technology for analysing histopathological images was summarised [1]. In order to evaluate the developed CAD systems, the authors additionally emphasised the usage of standard datasets because it makes analysis and comparison easier. Different approaches to the analysis of BCHI were reviewed by [8]. To increase the system’s robustness, the authors discussed how the complexity of the tissues’ unique properties necessitates further research [12]. The authors also examined how to approach using Machine Learning (ML) [13,14] for histopathological image analysis. A dataset available for the analysis of breast cancer and three generalised classification methods of images are used [15].

In [16] explored the different methods used to analyse histopathological images, such as nuclei detection, segmentation, feature extraction, and classification. A lack of standard datasets, difficulties relating to microscopic image segmentation, and problems involving ruggedness with references to clinical and technical conditions were also covered. His colleagues reviewed the most recent techniques for the segmentation of images used to extract features and classify diseases and discussed the features of images used in relation to histology. The computational procedures needed to detect cancer in histopathological images were reviewed by the authors. In their review, they looked into the different features that are used to diagnose various cancers.

In [17] investigated applying DL techniques in the field of digital pathology. Segmentation, detection, and classification use cases were the focus of this study. It has been proposed that combining the DL approach with hand-crafted features can enhance the quality of the classifiers. In [18], authors conducted a survey of the various image analysis techniques used in analysing histology images. The authors discussed issues related to cell detection and included issues that require resolution. The difficulties in the workflow of computational pathologies were reported by [19]. They discussed the potential lines of investigation for diagnostic [11] and provided a summary of cutting-edge techniques and usages in large-scale medical image analytics. The DL methods used in the field of medical image analysis were surveyed [20]. Modern DL techniques and difficulties encountered in the analysis of BCHI were discussed by the authors. The BreakHis dataset was used in [21] to conduct a thorough survey on the automatic diagnosis of breast cancer using DL techniques. In order to solve multi-category classification issues without regard to magnification, they also investigated the DL technique. The authors provided an overview of lymph node assistants for breast cancer images [22]. A thorough overview of the deep neural network architectures created to analyse histopathological images, as well as a list of problems and emerging trends, were presented in [23,24].

The development of CAD [25] systems was discussed, along with the ML and DL approaches used to detect breast carcinoma. They also examined various ML and DL methods for cancer diagnosis.

In [26] proposed a study for the classification of breast histopathological images, and for that, they suggested using the adopted patch selection approach on a smaller no. of training images and applying transfer learning. They explained that whole-slide images are used to extract the patches, and then feature extraction is undertaken by feeding them into a CNN. These features help us select the discriminative patches and then feed them into a pre-trained Efficient-Net architecture. The Support Vector Machine (SVM) classifier [27] is then used to train the features obtained from Efficient-Net. This model has proved to be better than the standard methods from the perspective of performance measures. In [28] introduced the residual convolution network developed using revolutionary AHoNet by jointly embedding statistical high order with attention mechanisms. To obtain local salient deep characteristics, AhoNet first uses an effective dimensionality deduction and local cross-platform interaction in a channel attention module. Then, matrix power normalisation further computes their second-order covariance statistics, resulting in a more reliable presentation of breast cancer as a global feature of pathology images. The experimental results show that AhoNet outperforms original single models in this clinical picture application, reaching the highest classification accuracy rates of 99.29% and 85% when used on the databases (BreakHis and BACH).

In [29] suggested a hybrid CNN [30] and RNN system for classifying images of breast cancer histopathology. Their approach combines the benefits of CNN and RNN and is built on a deeper multilevel feature extraction of the histopathological patches of images. Both the ST and LT spatial correlations between the patches are preserved. The research findings show that there is a 91.3% accuracy for the four-class classification. In, to resolve the two-class breast cancer classification in terms of pathology pictures, offered BiCNN, a revolutionary deep convolution-network-based breast cancer histopathological classification technique [31,32,33]. The category and subcategory labels for breast cancer are taken into account in this deep learning model as previous learning, which can limit the distance between the features in various cancer pathologies. [34] Additionally, a cutting-edge data augmentation approach that can fully preserve the image edge features of the cancerization zone is presented to limit the entire slide image classification. The research suggests the use of pre-trained models and a fine-tuning methodology to obtain the best results in terms of histopathology images for breast cancer. The findings of the experiment demonstrate that the suggested strategy shows a greater accuracy rate of 97% and exhibits stability and generalisation, providing effective tools for the medical detection of cancer.

In [35] presented a brand-new COVID-19 identification and classification system based on an unsupervised DL-based VAE (UDL-VAE). To improve the image quality, the UDL-VAE approach used an Adaptive Wiener Filtering (AWF) preparatory technique. Additionally, Inception v4 was used as a feature extractor with the Adagrad approach, and a non-teacher VAE model was used for classification. Several experiments were carried out to demonstrate the UDL-VAE model’s successful results in order to confirm its excellent diagnostic capability. With improved efficiencies of 0.987 and 0.992 for different distributions of classes (binary and multiple), accordingly, the acquired experimental values demonstrated the effective outcomes of the UDL-VAE model.

In [36] for better histological grading of breast cancer, an unique DL model was created and validated. The model leverages common histopathology pictures and has independent prognostic value for NHG 2 group classification. Molecular profiling is an expensive option, whereas model-based histology grading provides better risk classification at a lower cost. DeepGrade’s predictive value was subsequently evaluated in the external test set, which confirmed a higher risk of recurrence in DG2-high (HR 1.91, 95% CI 1.11–3.29, *p* = 0.019).

In [37] proposed a way to train the VAE model to make predictions on the image-based representation of the eye-tracking output. Their results showcased how the VAE model could generate good enough output from a limited dataset. This implies that VAE can be used for data augmentation tasks to increase overall performance in the classification tasks.

In their study [38], they proposed three effective EVAE-Net models for the detection of COVID-19. They trained two encoders on the images of chest X-rays to generate two feature maps. Their proposed model showed satisfactory performance, with the best model achieving 99.19% accuracy across four classes.

According to the literature, extensive research has been conducted in relation to automating histopathological image processing. However, there is not a thorough literature review that covers all facets of histopathological image analysis, such as colour normalisation, potential ROI detection and segmentation, feature extraction, and classification. Each of these review papers focuses on a specific facet of breast histopathological image analysis.

### Research Gaps

The first thing to note is that histopathology pictures of malignancy are fine-grained, elevated images that show intricate geometric patterns. Classification may be quite challenging, especially when dealing with several classes, because of the variety within a category and the similarity across classes [1]. The limits of feature extraction techniques for histopathology pictures of breast cancer present the second difficulty [39]. Scale-Invariant Feature Transform (SIFT) and Gray-Level Co-Occurrence Matrix (GLCM), two common approaches for extracting features, all depend on supervised data. The third difficulty in the field of tumour image processing is the annotated dataset, which is still a very difficult task [1]. Additionally, choosing valuable features requires previous information from the data, which lowers feature extraction efficiency and increases computing burden [40]. The final characteristics that were derived from the histopathology pictures are merely a few unimportant, low-level features. As a result, the final model may produce subpar categorization outcomes [8].

## 3. Proposed Approach

**Figure 1:**
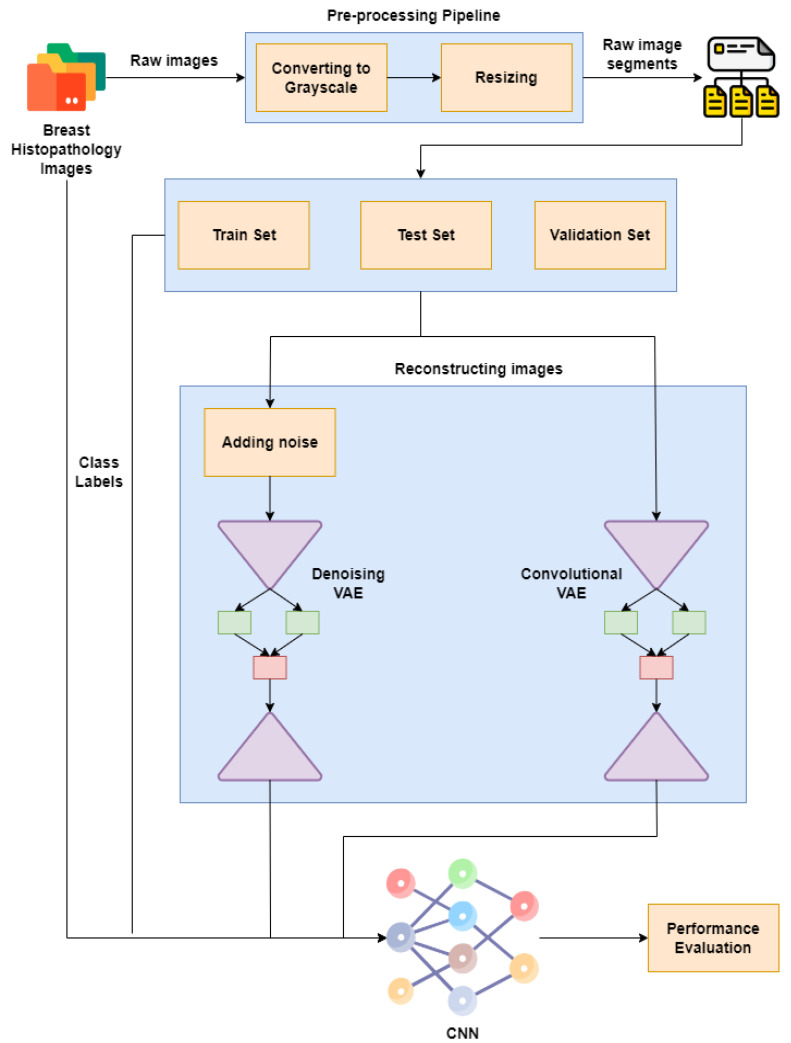
Proposed implementation pipeline.

**Figure 2:**
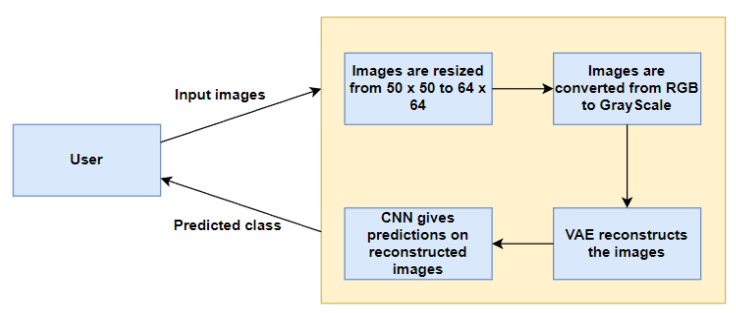
Data Flow Diagram.

**Figure 3:**
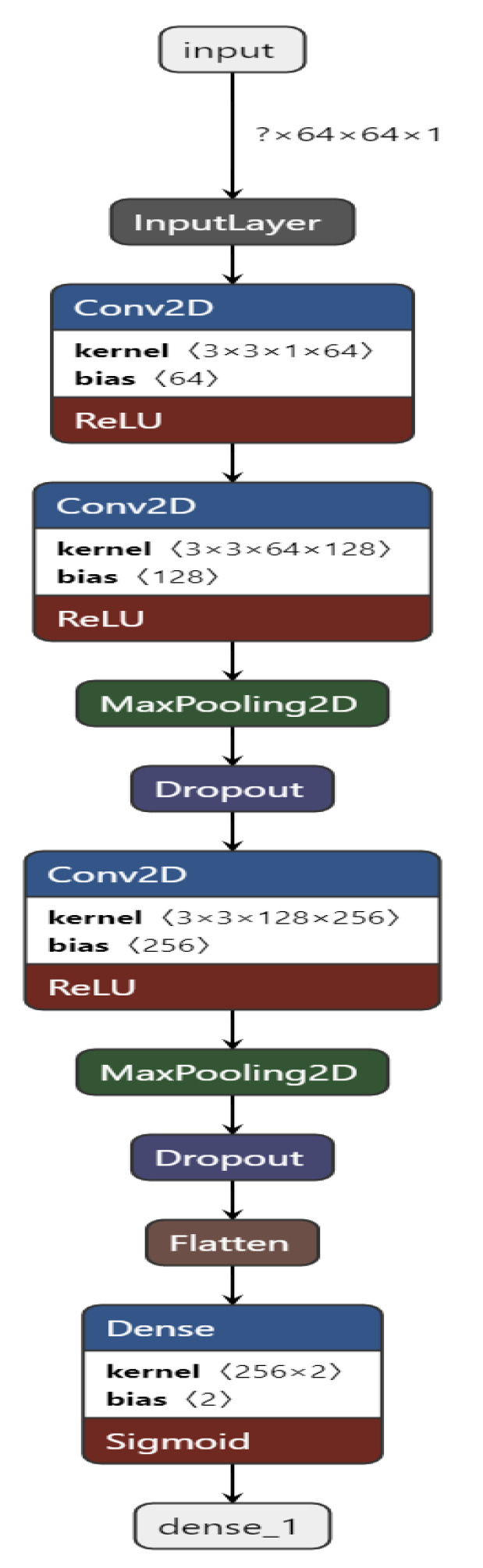
Convolutional Neural Network structure for the proposed work.

Figure 1 represents our proposed implementation pipeline, and in Figure 2, the data flow diagram for our proposed implementation pipeline is shown. We take 277,524 input images from our dataset [41]. The images are of 50 × 50 dimensions and in an RGB format. The dataset contains 198,738 images for IDC Negative and 78,786 images for IDC Positive. We choose 50,000 images in total from the dataset: 25,000 random images from both classes. The pre-processing stage includes converting the images to GrayScale and resizing them to 64 × 64. The input images are then normalised by dividing by 255. The dataset is split into train, test, and validation sets with sizes of 28,000, 10,000, and 12,000, respectively. To prove the usefulness of VAE, we first classify the images using a CNN to obtain a preliminary result. Figure 3 shows the neural network structure used for the Convolutional Neural Network. The loss function used is categorical cross entropy; the optimiser used is Adam, with a learning rate of 0.0005. The accuracy received is 69%.

We use two different kinds of VAE [42]: Convolutional VAE [43] and Denoising VAE. The Convolutional VAE of CVAE [44] comprises Convolutional layers as an encoder and decoder. The figure shows the basic structure of a VAE, and we use a similar one in our implementation. The latent dimension taken is 5, and the loss is calculated as the total of KL Divergence loss and reconstruction loss; the optimizer used is Adam [45], with a variable learning rate. The loss encountered is 41. The reconstructed images are then fed to the CNN used to achieve preliminary accuracy [46,47]. The CNN is trained on the reconstructed images, and the final accuracy received is 73%. The Denoising Variational Autoencoder DVAE is similar to CVAE with a slight difference; we add custom noise with a noise factor of 0.2 to the input images and then send the noisy input to the model for reconstruction. The loss function and the optimizer are also the same as that used in CVAE. The loss obtained is 43. The reconstructed images are then fed into the CNN used to obtain the preliminary accuracy. The CNN is trained on the reconstructed images, and the final accuracy received is 50%.

We discuss the Convolutional Neural Network, the VAE, and the DVAE individually in the below subsections.

### 3.1. Convolutional Neural Network (CNN)

**Figure 4:**
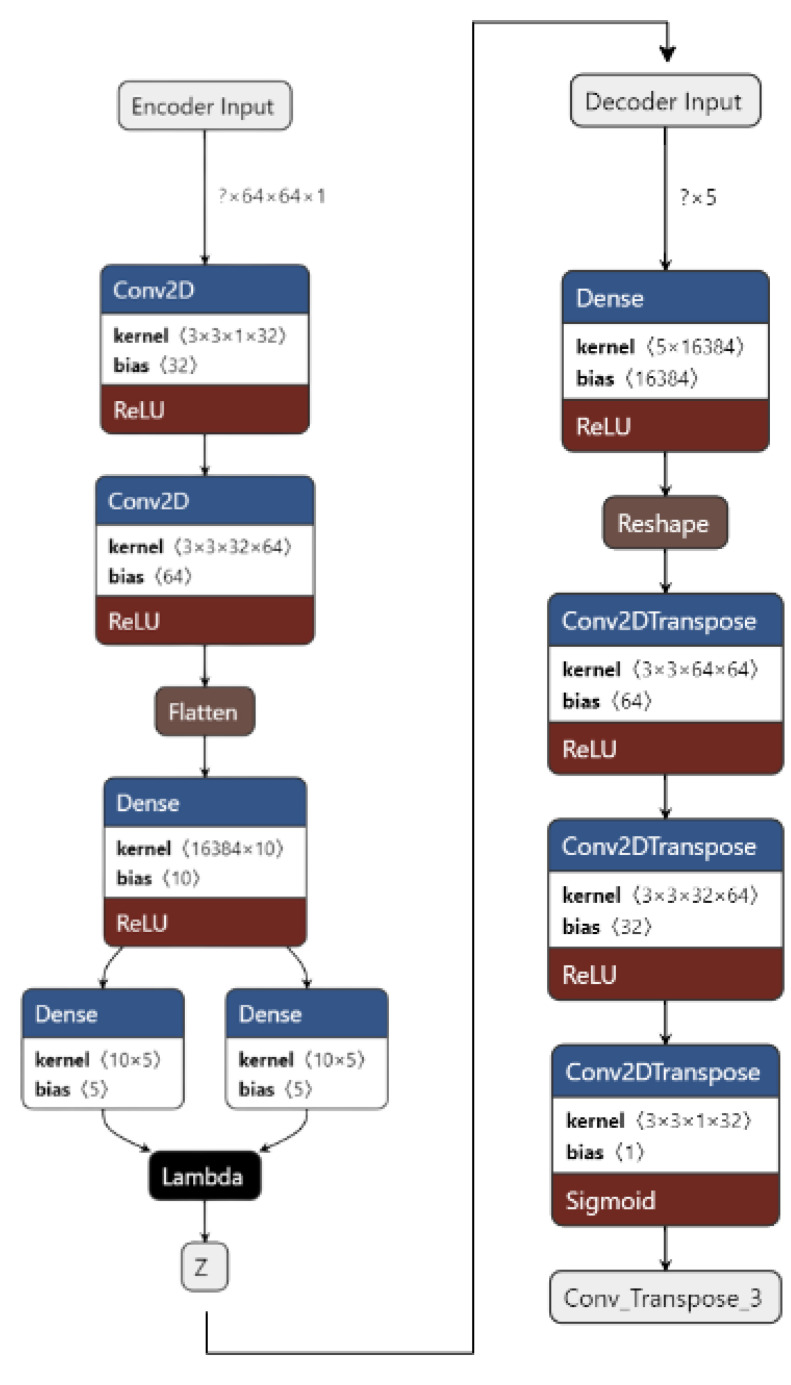
VAE for the proposed work.

Figure 4 shows the CNN structure used in our approach. In our approach, we built a CNN containing 10 layers, namely, 1 input layer, 3 Conv2D layers, 2 MaxPooling2D layers, 2 Dropout [48] layers, 1 Flatten layer, and 1 Dense output layer.

The CNN is built and trained using TensorFlow [49] and Keras [50]. The input layer takes in the image input with each image of shape = (64, 64, 1). All of the Conv2D layers have a kernel of size 3 and an activation function of ReLU.

The first Conv2D layer contains 64 filters, the second one has 128 filters, and the third one has 256 filters. The Dropout [48] layer drops 20% of the neurons, and the Dense layer has 2 units. The Convolutional and the Max Pooling layers successfully extract necessary features from the input image and provide predictions in the end after the Dropout [48] layer drops 20% of the neurons.

**Figure 5:**
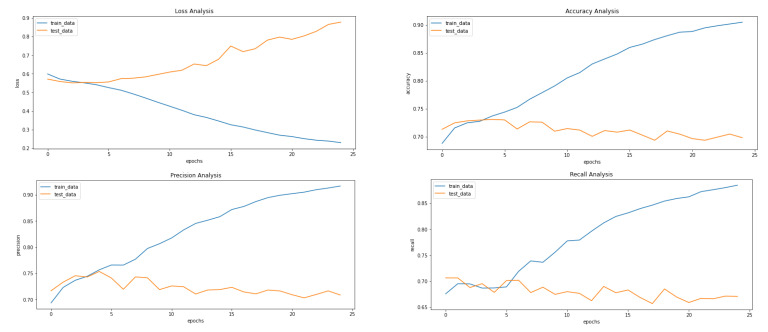
Results achieved after classification using CNN before applying VAE. It shows loss, accuracy, precision and recall from left to right.

The metrics used to evaluate the prediction are accuracy, loss, precision (see Equation (1)), recall (see Equation (2)), F1-Scode (see Equation (3)), specificity (see Equation (4)), Cohen’s Kappa (see Equation (5)), and ROC AUC, calculated while each epoch is training for the training set and after each epoch validation set. The loss used is categorical cross entropy (see Figure 5). The model is used to predict the classes of images before applying VAE to compare it with the results received after applying the VAE.

### 3.2. Variational Autoencoder

Figure 4 shows the implementation of VAE on our dataset. The input images are pre-processed. They are resized from 50 × 50 to 64 × 64 and converted from an RGB format to a GrayScale format using OpenCV. The images are then fed to the encoder. The encoder consists of 1 input layer, 2 Conv2D layers, 1 Flatten layer, 3 Dense layers, and an output Sampling layer. The input layer accepts the image input of the shape = (64, 64, 1). It forwards it to Conv2D layers which act as feature extractors. The features are then flattened in the flatten layer into 1D arrays. The flattened inputs are then fed to the dense layer, which then maps the flattened input vectors to mean and log variance and Dense layers, which are layers of the same number of units known as latent dimension. The latent dimension is the no. of units up to which the input vectors are encoded, and their dimension is reduced. The feature vectors are then forwarded to the Sampling layer to generate the probabilistic distribution, which will be fed to the decoder.

The decoder consists of 1 input layer, 1 dense layer, 1 reshape layer, and 3 Conv2Dtranspose layers. The input layer receives the input from the Sampling layer of the encoder of the shape = 16,384. The input is fed into the reshape layer to reshape the input vectors to the shape that was present before feeding the input into the Flatten layer. The reshaped vectors are then fed to the Conv2Dtranspose layers so that they can be converted into the original shape of (64, 64, 1).

The loss is calculated as the total of KL Divergence loss and Reconstruction loss. The data are now reconstructed and are ready to be fed into CNN to obtain the predictions.

### 3.3. Denoising Variational Autoencoder

**Figure 6:**
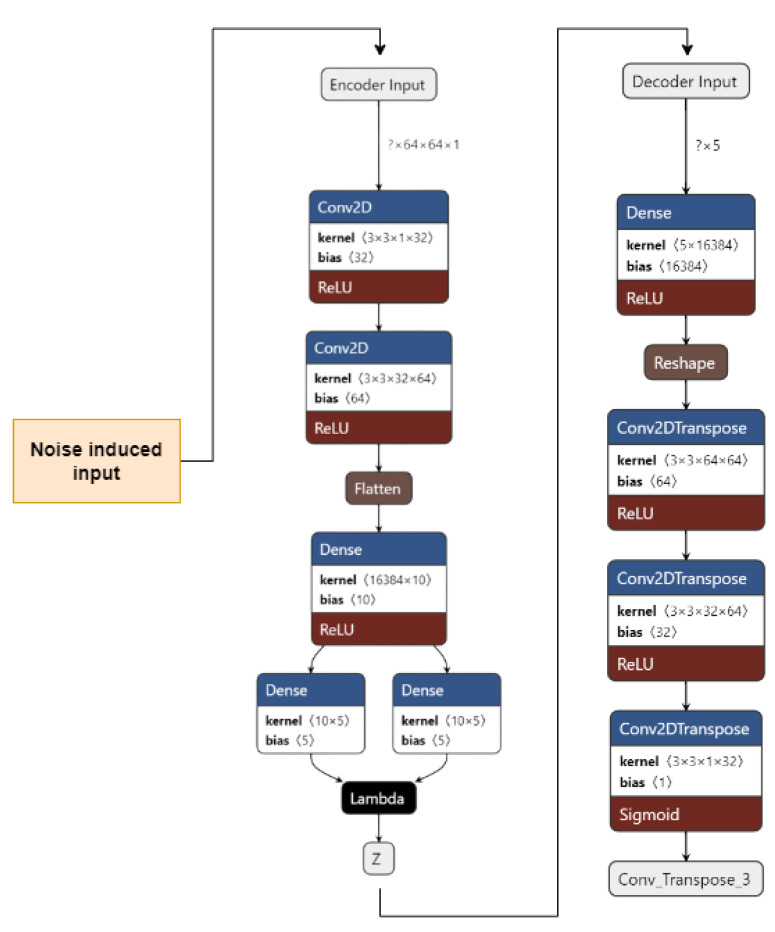
Denoising Variational Autoencoder for the proposed work.

Figure 6 shows our implementation of the DVAE used. The input images are pre-processed. They are resized from 50 × 50 to 64 × 64 and converted from an RGB format to a GrayScale format using OpenCV. A noise factor of 0.2 is induced into the images. The images are then fed to the encoder.

The encoder consists of 1 input layer, 2 Conv2D layers, 1 Flatten layer, 3 Dense layers, and an output Sampling layer. The input layer accepts the image input of the shape = (64, 64, 1). It forwards it to the Conv2D layers, which act as feature extractors. The features are then flattened in the Flatten layer into 1D arrays. The flattened inputs are then fed to the Dense layer, which then maps the flattened input vectors to mean and log variance; the Dense layers, which are layers of the same number of units known as the latent dimension. The latent dimension is the no. of units up to which the input vectors are encoded, and their dimension is reduced. The feature vectors are then forwarded to the Sampling layer to generate the probabilistic distribution, which will be fed to the decoder. The decoder consists of 1 input layer, 1 Dense layer, 1 Reshape layer, and 3 Conv2Dtranspose layers. The input layer receives input from the Sampling layer of the encoder of the shape = 16,384. The input is fed to the reshape layer to reshape the input vectors to the shape that was present before feeding the input into the Flatten layer. The reshaped vectors are then fed into the Conv2Dtranspose layers so that they can be converted into the original shape of (64, 64, 1).

The loss is calculated as the total of the KL Divergence loss and Reconstruction loss. The data are now reconstructed, the noise has been removed, and the data are now ready to be fed into CNN to obtain the predictions.

### 3.4. Dataset

Among breast cancer subtypes, invasive ductal carcinoma is the most prevalent. Pathologists frequently focus on areas that include the IDC when grading an entire mount sample’s aggressiveness. Therefore, properly identifying IDC zones inside a WSM is one of many pre-processing procedures for automatic aggressiveness rating.

**Figure 7:**
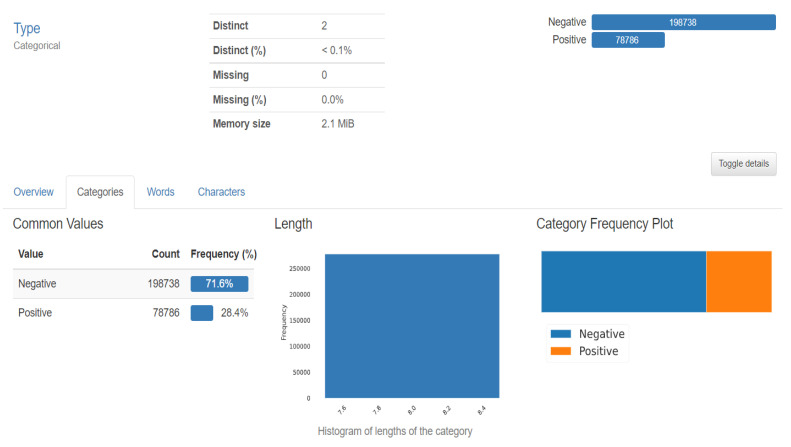
Dataset description.

The dataset included 162 images of WSM from Breast Cancer specimens that had been scanned at a magnification of 40× 50 × 50-inch patches measuring 277,524 in size were taken from that. The dataset used in the project is 5 years old and taken from Kaggle (Figure 7). The features of the datasets are mentioned in Table 1. There are 2 distinct classes, which symbolise IDC positive and IDC negative. There are 1,98,738 IDC negative images and 78,786 IDC positive images [18].

## 4. Experimental Setting and Result Analysis

The input images are resized to 64 × 64 from the original image size of 50 × 50. They are also converted into GrayScale from the original RGB colour scheme using OpenCV. The final shape of input images is (64, 64, 1).

The model is trained using GPU and default configurations provided by Google Colab and Kaggle notebooks. Hyperparameters configuration of the proposed classification models is shown in Table 2.

### Classification Metrics

Confusion matrix: Provides an understanding number for the classes that the model predicts properly and erroneously, and an error count is made. Recall, precision, specificity, and accuracy are some more metrics that can be measured with its help.

Precision: The proportion of positive predictions that came true is represented by precision (Equation (1)). As a result, it is also known as the positive predictive value.

Precision = TP/(TP + FP)(1)

Recall: Recall (Equation (2)) shows the percentage of positive samples that were correctly categorised. It also goes by the names sensitivity and true positive rate.

Recall = TP/(TP + FN)(2)

F1 Score: The harmonic mean of recall and precision is known as the F1 score (Equation (3)), and it is represented by the formula.

F1 Score = 2 × (Precision × Recall)/(Precision + Recall)(3)

Specificity: A test’s specificity (Equation (4)) is its capacity to label someone as negative for a disease even if they do not have it.

Specificity = TN/(FP + TN)(4)

Cohen’s Kappa: When two raters each assign N items to one of C mutually exclusive categories, the agreement between them is measured by Cohen’s kappa (Equation (5)).

Cohen’s Kappa = (P0 − Pe)/(1 − Pe)(5)

ROC AUC: The performance of a classification model at every classification threshold is depicted on a graph by the receiver operating characteristic curve, or ROC curve. Two variables are plotted on this curve:

True Positive Rate (Equation (6))
False Positive Rate (Equation (7))

True Positive Rate = FP/(FP + TN)(6)

False Positive Rate = TP/(TP + FN)(7)

**Figure 8:**
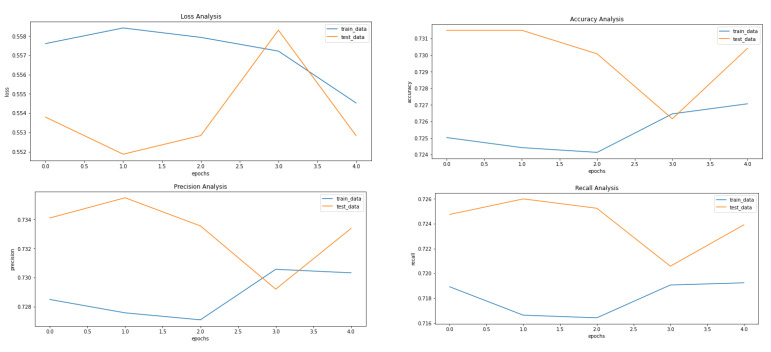
Results achieved after classification using CNN after applying and reconstructing input images using Convolutional VAE. It shows loss, accuracy, precision and recall from left to right.

**Figure 9:**
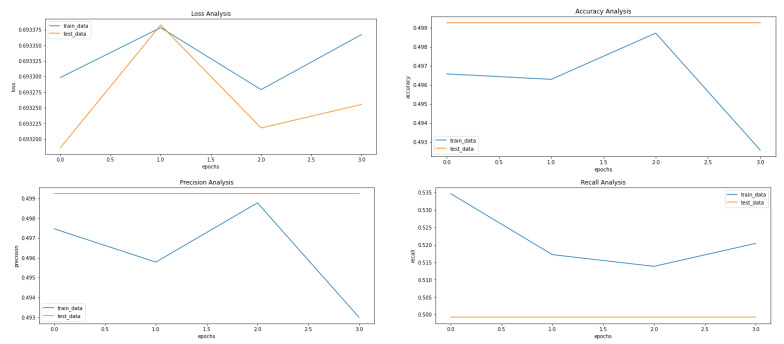
Results achieved after classification using CNN after applying and reconstructing input images using Denoising VAE. It shows loss, accuracy, precision and recall from left to right.

Figure 5, Figure 8 and Figure 9 showcase the Loss, Accuracy, Precision, and Recall, respectively. Based on the results achieved in Figure 8, the model is clearly overfitting on the dataset before applying VAE, which seems to be reduced in Figure 8, which showcases the results after applying VAE. Figure 9 shows the results after applying Denoising VAE, which are not up to the mark.

Table 3 shows the important metrics used to evaluate the proposed approach and proves that the application of VAE on our dataset proves to be fruitful with our proposed approach. The best results for the different measures are highlighted in Table 3. The final and best metrics are achieved after using VAE to reconstruct the images and then applying the CNN to classify the input image. The best accuracy achieved is 73%, while without using VAE, it comes out to be 68%.

Table 4 shows the comparative analysis between the various models implemented on our dataset. Pre-trained models EfficientNet B0 and EfficientNet B3 have been used for comparison along with the CNN model used in the proposed approach before implementing VAE.

## 5. Conclusions

In this work, we built a model based on deep learning for classifying input histopathological images of breast cancer. The dataset contains 277,524 image samples containing 198,738 IDC negative and 78,786 IDC positive 50 × 50 images. We obtained an accuracy of 0.6876 and an F1 Score of 0.6868 using a CNN.

After applying VAE, we obtained an accuracy of 0.7365 using the CNN after training it on the reconstructed dataset with an F1 Score of 0.7363, whereas after applying DVAE, we obtained an accuracy of 0.5002 using the CNN after training it on the reconstructed dataset with an F1 Score of 0.3335. The results obtained support our problem statement of enhancing breast cancer histopathology image analysis using VAE as the accuracy, and the F1 scores obtained before and after applying the VAE have a noticeable difference.

## Figures and Tables

**Table 1:** Dataset specification used for the proposed approach.

Property	Value
Number of samples	277,524
Type of image	RGB
Number of classes	2 (IDC positive and IDC negative)
Image size	50 × 50
Image magnification	40 times

**Table 2:** Hyperparameters configuration of the proposed classification models.

Model	Hyperparameters	Value
Convolutional Neural Network	Input shape (layer 1)	(64, 64, 1)
	Number of filters in Conv2D layers (layers 2–3, 6)	64, 128, 256
	Strides in Conv2D layers (layers 2–3, 6)	(2, 2)
	Kernel Size in Conv2D layers (layers 2–3, 6)	3
	Activation function in Conv2D layers (layers 2–3, 6)	Relu
	Pool size in MaxPool2D layers (layers 4, 7)	(1, 1)
	Dropout [48] rate in Dropout [48] layers (layers 5, 8)	0.2
	Units in Dense layer (layers 10)	2
	Activation function in Dense layer (10)	Sigmoid
Convolutional and Denoising Variational Autoencoder(Encoder)	Input shape (layer 1)	(64, 64, 1)
	Number of filters in Conv2D layers (layers 2–3)	32, 64
	Strides in Conv2D layers (layers 2–3)	2
	Kernel Size in Conv2D layers (layers 2–3)	3
	Activation function in Conv2D layers (layers 2–3)	Relu
	Padding in Conv2D layers (layers 2–3)	same
	Units in Dense layers (layers 5, 6–7)	10, 5
	Activation function used in Dense layer (layer 5)	Relu
	Units in Lambda layer (layer 9)	5
Convolutional and Denoising Variational Autoencoder(Decoder)	Input shape (layer 1)	5
	Units used in Dense layer (layer 2)	(16 × 16 × 64) = 16,384
	Target Shape used in Reshape layer (layer 3)	(16, 16, 64)
	Number of filters used in Conv2DTranspose layers (layers 4–6)	64, 32, 1
	Kernel size used in Conv2DTranspose layers (layers 4–6)	3
	Strides used in Conv2DTranspose layers (layers 4–6)	2, 2, 1
	Padding used in Conv2DTranspose layers (layers 4–6)	same
	Activation function used in Conv2DTranspose layers (layers 4–6)	Relu, Relu, Sigmoid

**Table 3:** Comparative analysis between classification results before and after implementing VAE. The best results are highlighted in bold.

Classifier	Metrics	Before VAE	After VAE	After DVAE
CNN	Loss	0.9449	0.5545	0.6932
CNN	Accuracy	0.6876	**0.7365**	0.5002
CNN	Precision	0.6805	0.7369	0.2502
CNN	Recall or Sensitivity	0.6984	0.7365	0.5002
CNN	F1 Score	0.6868	0.7363	0.3335
CNN	Specificity	0.69	0.74	0.50
CNN	Cohen’s Kappa	0.3749	0.4730	0.000
CNN	ROC AUC	0.7205	0.7969	0.5000

**Table 4:** Comparative analysis between classification results from different implementations including our best result. The best result is highlighted in bold.

Model	Result
CNN (without VAE)	Accuracy: 68%
EfficientNetB3	Accuracy: 69%
EfficientNetB0	Accuracy: 69%
CNN (with VAE)	**Accuracy: 73%**

## Data Availability

The data supporting the results were included in the article.
